# Editorial: Systemic, cross-sectoral, or regulatory interventions to improve population nutrition and related global health challenges

**DOI:** 10.3389/fpubh.2023.1336700

**Published:** 2023-12-20

**Authors:** Bai Li, Steve Allender, Wilma Waterlander

**Affiliations:** ^1^Centre for Exercise, Nutrition and Health Sciences, School for Policy Studies, University of Bristol, Bristol, United Kingdom; ^2^Global Obesity Centre (GLOBE), Institute for Health Transformation, Faculty of Health, Deakin University, Geelong, VIC, Australia; ^3^Public and Occupational Health, VUmc. De Boelelaan, Amsterdam, Netherlands

**Keywords:** systems approach, systems thinking, global health, malnutrition, obesity, reporting guidance, complexity

Malnutrition in all its forms is a leading cause for disability-adjusted life years (DALYs) globally (1). One in three people in the world suffers from at least one form of malnutrition, such as obesity or micronutrient deficiencies (2). Poor nutrition is driven by complex, interrelated environmental, social, cultural, political, economic, and behavioral factors (2). Regulatory and/or policy interventions across sectors are needed but face pushback from the system, i.e., vested interests (3). Methods from systems science have been advocated as useful tools to address this complexity and find sustainable solutions to malnutrition in all its forms (4). Although concepts and terminology of systems approaches have existed for many years, empirical knowledge about their application and effectiveness for public health nutrition remains very limited. Evidence is particularly lacking from low-and middle-income countries (LMICs). Uncertainty remains in terms of how an authentic systems approach can be applied in practice, how to engage non-academic partners – especially those who have the capacity and power to change health environments and policies – and how this relates to evidence standards.

In this special edition we sought contributions from international, national, and local health organizations, policymakers as well as academic authors working in population nutrition and related fields. It comprises 9 articles, representing contributions from 72 authors across institutions in 13 countries. The contributions provide insight into what these multiple partners are hoping to achieve from the application of systems approaches, how these projects might be conceived and presented as a research protocol, examples of their application in practice and proposed guidelines for the reporting of such studies.

Felmingham et al. reviewed the ways in which success has been characterized in the published literatures specifically around the use of system thinking in community prevention. The authors concluded that measures and concepts of success varied across the articles reviewed, ranging from level of community action, collaboration, changes in mental models, or cultural appropriateness, as well as shifts to a deeper understanding of complexity within the population. The article introduced a recurring theme throughout this special edition, which is the definition and measurement of success and the need for guidelines and standards in the design and reporting of such initiatives.

Examples from empirical studies using a systems approach are presented in the form of protocols or case studies of completed work. In the case of Speich et al. they presented a research protocol for the development of projects targeting governance, policy and supply to improve food and nutrition security in several “secondary” cities in low income countries. They took a systems approach by proposing the development of a transdisciplinary intervention drawing on agriculture, food and health sectors to improve value chains with respect to six specific cities in Bangladesh, Kenya and Rwanda. In addition, they proposed working from a theory of change and focusing on elements of policy and advocacy, building of institutional capacity, data-driven planning and resource mobilization, workforce development and provision of feedback loops to support ongoing implementation.

A study by Allender et al. used participatory research methods in a co-creation study for enhancing policy to address diabetes in the Indian Ocean territories, a remote set of islands between Australia and Indonesia. The process itself used group model building (GMB), a technique prominent in systems science. The study provides an insight into adaptations required to such projects arising from travel restrictions during the COVID pandemic. Community perceptions were collected using methods from systems science and views were sought from a wide range of stakeholders across the islands. Participants described the systemic drivers of diabetes on the islands and potential policy solutions ranging from freight cost to food policy.

Work set in South Carolina (Calancie et al.) also brought multiple stakeholders together, using GMB to explicitly understand and intervene in the systemic drivers of child obesity. The participatory approach led to a range of priorities for interventions across multiple system levels, including food insecurity, empowering minority populations and advocacy for change across all sectors of the community.

Endevelt et al. called for better engagement with key stakeholders in design and implementation whether the context be LMIC or high-income countries. Their case study of attempts to implement policies for fortification of food in Israel emphasized the need for rigorous and structured engagement of all stakeholders and clear mechanisms for knowledge exchange across all levels to achieve optimal systems change. Key to this is capturing and sharing how these processes work, and what makes them effective, to create generalisable models for use worldwide.

Two studies addressed aspects of this multi-persepctive challenge in particularly in understanding the complexity of obesity and diabetes in Amsterdam and Qatar. Pinzon et al. aimed to identify and comprehend the fundamental system dynamics influencing obesity-related behaviors among adolescents. To achieve this, they constructed a Causal Loop Diagram (CLD) from a multi-actor viewpoint and then conducted a systems-based analysis to gain insights into the existing system, considering both its structural and functional aspects. The CLD presented in this study represented a synthesis of insights from academic researchers, adolescents, and stakeholders. Notably, adolescents made the most substantial contribution to the CLD, accounting for 74 out of 121 factors. A key finding was the ways in which existing structure worked to promote unhealthy behaviors among adolescents. When examining the emergent properties of the system from a macro perspective, it became evident that the functioning of several subsystems was oriented toward the objective of optimizing short- or long-term economic growth within the framework of a market-driven economy.

Analysis by Alareeki et al. developed deterministic models of public health interventions regarding the burden of diabetes burden among Qatari adults. The approach built on a mathematical model of the complexity of interacting modifiable and non-modifiable risks to assess the impact of a range of public health interventions, from lifestyle intervention to policy changes for active transport and in support of healthier food systems. A key finding was that multiple interventions at both individual and structural levels would deliver a greater impact than single interventions acting within one system alone.

Across this series, there are several commonalities, notably a focus on designing interventions that will have an impact and that will actively engage with the need for systemic action operating at multiple levels of risk and benefit. A second theme is trying to understand and engage the mechanisms by which interventions work or fail with the goal of identifying an optimal mix of interventions within a complex environment to provide the best return on investment. A third theme is the importance of putting key actors (e.g., community, healthcare professionals, educators, researchers, retailers, adolescents, etc) at the center of the design process: recognizing that change in these complex systems requires active engagement and co-creation with those who live and work within them.

A review by Li, Alharbi et al. found very few studies could claim rigid adherence to application of systems thinking or methods at all stages of the process, and the included studies were all conducted in high-income countries. Common features shared by the included studies were identified, such as measuring ongoing changes, in addition to endpoint outcomes, and supporting capacity building. Sub-optimal reporting might have explained the small number of studies meeting inclusion criteria, so Li, Allender et al. developed a list of practical questions (reporting guidance) to assist academic authors, journal editors and other interested stakeholders to design, report or review future interventions that apply a systems approach to tackle obesity or other public health challenges. These questions were developed based on the latest academic knowledge and are organized by the three broadly defined and interrelated stages of an intervention's life cycle: “development,” “implementation/delivery” and “evaluation.” The reporting guidance recognizes that in practice, the process of developing, implementing/delivering, and evaluating any complex intervention is often iterative and reflective, providing room for the main stages of the intervention's life cycle to occur simultaneously.

In summary, this Research Topic demonstrates a growing and comprehensive application of systems thinking principles in public health research. However, there is a pressing need for clearer definitions and better reporting of these approaches. We recommend that journals and authors adopt such standards, similar to those used for other methodologies such as randomized controlled trials (RCTs) or systematic reviews.

## Author contributions

BL: Conceptualization, Formal analysis, Project administration, Resources, Visualization, Writing—original draft, Writing—review & editing. SA: Conceptualization, Formal analysis, Project administration, Resources, Visualization, Writing—original draft, Writing—review & editing. WW: Conceptualization, Formal analysis, Project administration, Resources, Visualization, Writing—original draft, Writing—review & editing.

## References

[1] Murray CJ Aravkin AY Zheng P Abbafati C Abbas KM Abbasi-Kangevari M. Global burden of 87 risk factors in 204 countries and territories, 1990–2019: a systematic analysis for the global burden of disease study 2019. Lancet. (2020) 396:1223–49. 10.1016/S0140-6736(20)30752-2 33069327 PMC7566194

[2] Global Nutrition Report. Global Nutrition Report: Stronger Commitments for Greater Action. Bristol: Development Initiatives (2022).

[3] Swinburn B. Power dynamics in 21st-century food systems. Nutrients. (2019) 11:2544. 10.3390/nu11102544 31652523 PMC6835350

[4] Li B He Z Peters R Allender S Zou Y Zhou W. Cultural adaptations and methodological innovations to group model building for the systems actions to reduce malnutrition in all its forms in Southeast Asian countries and China (SYSTAM CHINA-SEACS international consortium) project. Int J Behav Nutr Phys Activity. (2023) 20:111. 10.1186/s12966-023-01510-5 37723534 PMC10506199

